# Direction of Arrival Estimation of Generalized Nested Array via Difference–Sum Co-Array

**DOI:** 10.3390/s23020906

**Published:** 2023-01-12

**Authors:** Yule Zhang, Guoping Hu, Hao Zhou, Juan Bai, Chenghong Zhan, Shuhan Guo

**Affiliations:** 1 Graduate College, Air Force Engineering University, Xi’an 710051, China; 2 Air and Missile Defense College, Air Force Engineering University, Xi’an 710051, China

**Keywords:** direction of arrival estimation, sparse array, generalized nested array, difference–sum co-array, degrees of freedom, atomic norm

## Abstract

To address the weakness that the difference co-array (DCA) only enhances the degrees of freedom (DOFs) to a limited extent, a new configuration called the generalized nested array via difference–sum co-array (GNA-DSCA) is proposed for direction of arrival (DOA) estimation. We consider both the temporal and spatial information of the array output to construct the DSCA model, based on which the DCA and sum co-array (SCA) of the GNA are systematically analyzed. The closed-form expression of the DOFs for the GNA-DSCA is derived under the determined dilation factors. The optimal results show that the GNA-DSCA has a more flexible configuration and more DOFs than the GNA-DCA. Moreover, the larger dilation factors yield significantly wider virtual aperture, which indicates that it is more attractive than the reported DSCA-based sparse arrays. Finally, a hole-filling strategy based on atomic norm minimization (ANM) is utilized to overcome the degradation of the estimation performance due to the non-uniform virtual array, thus achieving accurate DOA estimation. The simulation results verify the superiority of the proposed configuration in terms of virtual array properties and estimation performance.

## 1. Introduction

Direction of arrival (DOA) estimation has increasingly become a hot topic in the field of array signal processing since it is continually encountered in many applications, such as radar, wireless communication and automatic driving [1,2,3,4,5,6,7,8,9,10,11,12,13]. In the past half century, numerous subspace-based algorithms [10,11] and sparse reconstruction approaches [12,13] have been proposed to estimate the incident direction of multiple sources. It is well known that, for an N-sensor uniform linear array (ULA), the above methods can only identify up to N−1 sources. Therefore, additional sensors are required in the ULA to achieve the desired estimation performance, which dramatically increases the system’s overhead and hardware costs. Nevertheless, in practical scenarios, such as unmanned driving and 5G communication, the number of sources is greater than that of sensors. Consequently, the contradiction between economic cost and realistic demand leads to underdetermined DOA estimation as a very tricky problem.

To tackle this deficiency, a Khatri–Rao subspace approach was reported in [14], thus 2N−2 sources can be resolved by an N-sensor ULA. The nested array (NA) [15] with $N_{1}+N_{2}$ sensors was subsequently proposed in conjunction with the Khatri–Rao product to provide $2N_{2}\left(N_{1}+1\right)-1$ degrees of freedom (DOFs), which significantly increases the number of estimable sources. Moreover, the same process was applied to the coprime array (CPA) [16] with M+N−1 sensors to generate MN+M+N−2 DOFs, where M and N are a pair of integers. Inspired by the Khatri–Rao concept, extensive efforts have been devoted to designing sensor arrays with more DOFs to improve the estimation performance. At present, various new sparse arrays have been derived from NA and CPA, including the super nested array [17,18], augmented nested array [19], generalized nested array (GNA) [20], generalized coprime array [21], thinned coprime array [22], padded coprime array [23], enhanced and generalized sparse arrays [24,25,26], and coprime planar array [27], etc. Unfortunately, some sparse arrays designed to suppress the mutual coupling have a non-uniform difference co-array (DCA), indirectly degrading the performance of the co-array subspace-based algorithms [28,29]. To fully extract all the information contained in the non-uniform virtual array, a sparsity-based DOA estimation method based on the Least Absolute Shrinkage and Selection Operator (LASSO) was proposed in [30], which offers more DOFs than the co-array MUSIC algorithm [28]; yet, the predefined spatial grids result in basis mismatch or enormous complexity. To cope with both the information loss and the high computation requirements, a multifrequency method [31] was proposed to fill the holes in the DCA, and thus all DOFs can be utilized to perform spectra estimation, whereas it requires the sources to have sufficient bandwidth. Most recently, a class of DOA estimation algorithms for coprime arrays via virtual array interpolation [32,33,34,35,36] has triggered wide concern because, in contrast to [30], these approaches do not need to predefine the sampling grids. In [32], the nuclear norm minimization and the matrix completion principle were developed to recover the missing lags at the hole locations; thus, the virtual array is directly expanded to a continuous ULA. Furthermore, the minimum number of virtual arrays required to accomplish the matrix completion was defined in [33]. In [35], the restoration problem of the interpolated virtual sensors is solved by minimizing the atomic norm of the observation vector.

Although the aforementioned sparse arrays and DOA estimation algorithms can identify more sources than the number of physical sensors, only the DCA is considered, while the information contained in the sum co-array (SCA) is completely ignored. However, active sensing shows that the SCA has the capacity to further enhance the DOFs [37,38,39]. With that in mind, a vectorized conjugate-augmented MUSIC estimator for a CPA was presented in [40] by using both the temporal and spatial information of the array output to construct the difference–sum co-array (DSCA), providing more DOFs than the DCA. Following this concept, the DSCA properties of the NA were analyzed in [41], based on which a novel configuration called the diff–sum nested array (DsNA) was proposed. The DsNA is obtained by moving nearly half of the dense sensors in the NA to the right area of the sparse ULA, yielding a larger virtual aperture, while reducing the redundant lags. In [42], the concept of a translation nested array (TNA) was defined. Then, two improved nested arrays (INAwSDCA−I and INAwSDCA−II) with more consecutive DOFs were derived by rearranging the sensor positions of the TNA.

To alleviate the mutual coupling, while increasing the DOFs, we develop the GNA via the DSCA (GNA-DSCA) for DOA estimation. Different to the GNA-DCA [20], which only exploits the spatial information, the GNA-DSCA utilizes both the temporal and spatial information to obtain the delay correlations. Furthermore, the additional information is contained in the sum co-array. Therefore, compared with the GNA-DCA, the GNA-DSCA is an improved method which can form the difference–sum co-array. Specifically, the closed-form expression of the DOFs is derived, which can be expressed as a function of the dilation factors and the number of sensors. The best results show excellent properties as follows: (1) Compared with the GNA-DCA [20], the GNA-DSCA can pick more dilation factors and thus has more flexible configurations. (2) The GNA-DSCA provides more consecutive lags with about twice the maximum DOFs as the GNA-DCA. (3) The GNA-DSCA has a larger virtual array aperture than the existing DSCA-based sparse arrays, including the CPA-DSCA [40], DsNA [41], INAwSDCA−I [42] and INAwSDCA−II [42]. Then, a hole-filling strategy based on atomic norm minimization (ANM) is elaborated, with the aim of overcoming the discontinuity of the virtual domain. Based on the reconstructed Toeplitz matrix, we achieve accurate DOA estimation for the GNA-DSCA.

Notations: Matrices and vectors are represented by upper-case italic bold letters (e.g., A) and lower-case italic bold letters (e.g., a). In particular, $I_{M}$ is the M×M identity matrix. The superscripts ${\left(\cdot \right)}^{T}$, ${\left(\cdot \right)}^{*}$ and ${\left(\cdot \right)}^{H}$ represent the transpose, conjugate and conjugate–transpose operations, respectively. $J=\sqrt{-1}$ and δ(·) imply the imaginary unit and Dirac delta. diag(a) denotes the diagonal matrix formed using the entries of a. The symbols ∘, ⊗ and ⊕ denote the Khatri–Rao product, Kronecker product and Hadamard product, respectively. vec(A) represents the vectorization operator that turns A into a vector by stacking each column in sequence. |A| is the cardinality of an integer set A. The atomic norm and Frobenius norm are written as ${\left\|\cdot \right\|}_{A}$ and ${\left\|\cdot \right\|}_{F}$, respectively. rank(A) and trace(A) denote the trace and rank of A. We also define n∈〈a,b〉, such that n denotes any integer from the integers a to b.

## 2. Signal Model

Assume K far-field, narrowband and uncorrelated sources with a wavelength λ impinging on the G-sensor sparse linear array from directions $\Theta =\left\{\theta_{k}|k=1,2,\cdots ,K\right\}$, where $\theta_{k}\in \left[-\pi /2,\pi /2\right)$. The sensor locations $p_{m}$ are presented as the integer set $\mathbb{P}=\left\{p_{m}|m\in \langle 1,G\rangle \right\}$, in which all sensor positions are normalized by the unit inter-sensor spacing d=λ/2 and the first sensor is chosen as a reference, i.e., $p_{1}=0$. The array output vector, at time t, is modeled as

(1) $$x(t)=\sum_{k=1}^{K}a\left(\theta_{k}\right)s_{k}\left(t\right)+n\left(t\right)=As\left(t\right)+n\left(t\right)$$

where $A=\left[a\left(\theta_{1}\right),a\left(\theta_{2}\right),\cdots ,a\left(\theta_{K}\right)\right]$ behaves like the array manifold, with $a\left(\theta_{k}\right)={\left[1,e^{-J2\pi p_{2}\sin\theta_{k}/\lambda },\cdots ,e^{-J2\pi p_{G}\sin\theta_{k}/\lambda }\right]}^{T}$ denoting the steering vector of the k-th source. $s\left(t\right)={\left[s_{1}\left(t\right),s_{2}\left(t\right),\cdots ,s_{K}\left(t\right)\right]}^{T}$ refers to the source vector with $s_{k}\left(t\right)=u_{k}e^{J\left(\omega +\omega_{k}\right)t}$, where ω is the carrier frequency, and $u_{k}$ and $\omega_{k}$ denote the complex amplitude and a small frequency offset, respectively. After demodulation to IF, the k-th signal becomes $s_{k}\left(t\right)=u_{k}e^{J\omega_{k}t}$. $n\left(t\right)={\left[n_{1}\left(t\right),n_{2}\left(t\right),\cdots ,n_{G}\left(t\right)\right]}^{T}∼CN\left(0,\sigma_{n}^{2}I_{G}\right)$ is assumed to be the observed white Gaussian noise vector and uncorrelated with s(t).

Let $x_{m}\left(t\right)$ denote the m-th sensor output and τ denote the time lag. By collecting $N_{s}$ snapshots, the time average function of $x_{1}^{*}\left(t\right)$ and $x_{m}\left(t+\tau \right)$ is formulated by

(2) $$\begin{array}{ll} R_{x_{1}^{*}x_{m}}\left(\tau \right) & =\frac{1}{N_{s}}\sum_{t=1}^{N_{s}}x_{1}^{*}\left(t\right)x_{m}\left(t+\tau \right)\\ & =\frac{1}{N_{s}}\sum_{t=1}^{N_{s}}\left\{{\left[\sum_{k=1}^{K}a_{1}\left(\theta_{k}\right)s_{k}\left(t\right)+n_{1}\left(t\right)\right]}^{*}\times \left[\sum_{q=1}^{K}a_{m}\left(\theta_{q}\right)s_{q}\left(t+\tau \right)+n_{m}\left(t+\tau \right)\right]\right\}\\ & =\sum_{k=1}^{K}\sum_{q=1}^{K}\left\{a_{1}^{*}\left(\theta_{k}\right)a_{m}\left(\theta_{q}\right)\frac{1}{N_{s}}\sum_{t=1}^{N_{s}}s_{k}^{*}\left(t\right)s_{q}\left(t+\tau \right)\right\}+\frac{1}{N_{s}}\sum_{t=1}^{N_{s}}n_{1}^{*}\left(t\right)n_{m}\left(t+\tau \right)\\ & =\sum_{k=1}^{K}\sum_{q=1}^{K}\left\{a_{1}^{*}\left(\theta_{k}\right)a_{m}\left(\theta_{q}\right)u_{k}^{*}u_{q}e^{J\omega_{q}\tau }\frac{1}{N_{s}}\sum_{t=1}^{N_{s}}e^{J\left(\omega_{q}-\omega_{k}\right)t}\right\}+R_{n_{1}^{*}n_{m}}\left(\tau \right) \end{array}$$

where $R_{n_{1}^{*}n_{m}}\left(\tau \right)=\sigma_{n}^{2}\delta \left(\tau \right)\delta \left(m-1\right)$. If τ≠0, we can obtain $R_{n_{1}^{*}n_{m}}\left(\tau \right)=0$. Note that in the case $N_{s}$ is adequately large and $\omega_{q}\neq \omega_{k}$, one has $\sum_{t=1}^{N_{s}}e^{J\left(\omega_{q}-\omega_{k}\right)t}/N_{s}\approx 0$. Thereby, Equation (2) can be simplified as

(3) $$R_{x_{1}^{*}x_{m}}\left(\tau \right)=\sum_{k=1}^{K}\left\{a_{1}^{*}\left(\theta_{k}\right)a_{m}\left(\theta_{k}\right)\frac{1}{N_{s}}\sum_{t=1}^{N_{s}}s_{k}^{*}\left(t\right)s_{k}\left(t+\tau \right)\right\}=\sum_{k=1}^{K}e^{-J2\pi p_{m}\sin\theta_{k}/\lambda }R_{s_{k}^{*}s_{k}}\left(\tau \right)$$

where $R_{s_{k}^{*}s_{k}}\left(\tau \right)=\sum_{t=1}^{N_{s}}s_{k}^{*}\left(t\right)s_{k}\left(t+\tau \right)/N_{s}={\left|u_{k}\right|}^{2}e^{J\omega_{k}\tau }$ has a similar form to the incident source $s_{k}\left(t\right)=u_{k}e^{J\omega_{k}t}$; thus, it can be regarded as an equivalent source from $\theta_{k}$ with power ${\left|u_{k}\right|}^{4}$.

Combining all the $R_{x_{1}^{*}x_{m}}\left(\tau \right)$ into a column vector yield

(4) $$R_{x}\left(\tau \right)=AR_{s}\left(\tau \right)$$

where $R_{x}\left(\tau \right)={\left[R_{x_{1}^{*}x_{1}}\left(\tau \right),R_{x_{1}^{*}x_{2}}\left(\tau \right),\cdots ,R_{x_{1}^{*}x_{G}}\left(\tau \right)\right]}^{T}$ and $R_{s}\left(\tau \right)={\left[R_{s_{1}^{*}s_{1}}\left(\tau \right),R_{s_{2}^{*}s_{2}}\left(\tau \right),\cdots ,R_{s_{K}^{*}s_{K}}\left(\tau \right)\right]}^{T}$.

Replacing the independent variable τ in Equation (4) to −τ and taking the conjugate, one can obtain the following result

(5) $$R_{x}^{*}\left(-\tau \right)=A^{*}R_{s}^{*}\left(-\tau \right)=A^{*}R_{s}\left(\tau \right)$$

Then, we stack $R_{x}\left(\tau \right)$ and $R_{x}^{*}\left(-\tau \right)$ to construct the conjugate-augmented correlation vector R(τ) as follows

(6) $$R\left(\tau \right)=\left[\begin{array}{c} R_{x}^{*}\left(-\tau \right)\\ R_{x}\left(\tau \right) \end{array}\right]=\left[\begin{array}{c} A^{*}\\ A \end{array}\right]R_{s}\left(\tau \right)$$

Assigning the appropriate pseudo-sampling period $T_{p}$ and the pseudo-sampling snapshot $N_{p}$ to the independent variable τ, we can obtain the pseudo-data matrix Y as follows

(7) $$Y=\left[R\left(T_{p}\right),R\left(2T_{p}\right),\cdots ,R\left(N_{p}T_{p}\right)\right]=BUE$$

where

(8) $$E=\left[\begin{array}{cccc} e^{J\omega_{1}T_{p}} & e^{J\omega_{1}2T_{p}} & \cdots & e^{J\omega_{1}N_{P}T_{p}}\\ e^{J\omega_{2}T_{p}} & e^{J\omega_{2}2T_{p}} & \cdots & e^{J\omega_{2}N_{P}T_{p}}\\ \vdots & \vdots & \vdots & \vdots \\ e^{J\omega_{K}T_{p}} & e^{J\omega_{K}2T_{p}} & \cdots & e^{J\omega_{K}N_{P}T_{p}} \end{array}\right]$$

$B=\left[b\left(\theta_{1}\right),b\left(\theta_{2}\right),\cdots ,b\left(\theta_{K}\right)\right]$ with the k-th column being $b\left(\theta_{k}\right)={\left[a^{H}\left(\theta_{k}\right),a^{T}\left(\theta_{k}\right)\right]}^{T}$, and $U=diag\left(\left[{\left|u_{1}\right|}^{2},{\left|u_{2}\right|}^{2},\cdots ,{\left|u_{K}\right|}^{2}\right]\right)$.

By collecting $N_{p}$ pseudo-sampling snapshots, the covariance matrix of Y can be calculated as

(9) $$R_{Y}=\frac{1}{N_{p}}YY^{H}=BU\left(\frac{1}{N_{p}}EE^{H}\right)U^{H}B^{H}$$

where

(10) $$\frac{1}{N_{p}}EE^{H}=\left[\begin{array}{cccc} 1 & \sum_{n=1}^{N_{p}}\frac{e^{J\left(\omega_{1}-\omega_{2}\right)nT_{p}}}{N_{p}} & \cdots & \sum_{n=1}^{N_{p}}\frac{e^{J\left(\omega_{1}-\omega_{K}\right)nT_{p}}}{N_{p}}\\ \sum_{n=1}^{N_{p}}\frac{e^{J\left(\omega_{2}-\omega_{1}\right)nT_{p}}}{N_{p}} & 1 & \cdots & \sum_{n=1}^{N_{p}}\frac{e^{J\left(\omega_{2}-\omega_{K}\right)nT_{p}}}{N_{p}}\\ \vdots & \vdots & \vdots & \vdots \\ \sum_{n=1}^{N_{p}}\frac{e^{J\left(\omega_{K}-\omega_{1}\right)nT_{p}}}{N_{p}} & \sum_{n=1}^{N_{p}}\frac{e^{J\left(\omega_{K}-\omega_{2}\right)nT_{p}}}{N_{p}} & \cdots & 1 \end{array}\right]$$

When $N_{p}$ is adequately large, we have $\sum_{n=1}^{N_{p}}e^{J\left(\omega_{2}-\omega_{1}\right)nT_{p}}/N_{p}\approx 0$. Thus, Equation (10) can be approximated as

(11) $$\frac{1}{N_{p}}EE^{H}\approx I_{K}$$

As a result, Equation (9) can be reformulated as

(12) $$R_{Y}=BVB^{H}$$

where $V=UI_{K}U^{H}=\mathrm{diag}\left(\left[{\left|u_{1}\right|}^{4},{\left|u_{2}\right|}^{4},\cdots ,{\left|u_{K}\right|}^{4}\right]\right)$.

Vectorizing $R_{Y}$ yields

(13) $$\begin{array}{cc} r & =\mathrm{vec}\left(R_{Y}\right)=\mathrm{vec}\left(BVB^{H}\right)\\ & =\sum_{k=1}^{K}{\left|u_{k}\right|}^{4}\mathrm{vec}\left(b\left(\theta_{k}\right)b^{H}\left(\theta_{k}\right)\right)\\ & =\left(B^{*}\circ B\right)u=Cu \end{array}$$

where $u={\left[{\left|u_{1}\right|}^{4},{\left|u_{2}\right|}^{4},\cdots ,{\left|u_{K}\right|}^{4}\right]}^{H}$. The k-th column of $C=B^{*}\circ B$ can be expressed as

(14) $$b^{*}\left(\theta_{k}\right)⊗b\left(\theta_{k}\right)={\left[\begin{array}{c} a^{*}\left(\theta_{k}\right)\\ a\left(\theta_{k}\right) \end{array}\right]}^{*}⊗\left[\begin{array}{c} a^{*}\left(\theta_{k}\right)\\ a\left(\theta_{k}\right) \end{array}\right]=\left[\begin{array}{c} a\left(\theta_{k}\right)⊗a^{*}\left(\theta_{k}\right)\\ a\left(\theta_{k}\right)⊗a\left(\theta_{k}\right)\\ a^{*}\left(\theta_{k}\right)⊗a^{*}\left(\theta_{k}\right)\\ a^{*}\left(\theta_{k}\right)⊗a\left(\theta_{k}\right) \end{array}\right]=\left[\begin{array}{c} diff1\\ sum1\\ sum2\\ diff2 \end{array}\right]$$

where $diff1=e^{-J2\pi \left(p_{m}-p_{n}\right)\sin\theta_{k}/\lambda }$, $sum1=e^{-J2\pi \left(p_{m}+p_{n}\right)\sin\theta_{k}/\lambda }$, $sum2=e^{-J2\pi \left(-p_{m}-p_{n}\right)\sin\theta_{k}/\lambda }$ and $diff2=e^{-J2\pi \left(-p_{m}+p_{n}\right)\sin\theta_{k}/\lambda }$. It is obvious that the virtual sensor positions in C are collected as the difference–sum co-array, which is the union set of the difference co-array and the sum co-array. The exact descriptions of them are given in Section 3.

## 3. Generalized Nested Array

We start by introducing the following terminologies to facilitate the description of the physical configuration and the corresponding virtual array characteristics in the sequel. Hereafter, the sensor locations are normalized by the underlying inter-sensor spacing d.

**Definition 1.**  *(Set Operations). For two given integer sets* $X_{1}$*and*$X_{2}$*, while denoting* $X=X_{1}\cup X_{2}$*, we can define the following operations:*

(a) *Self-difference*: $D_{X-sd}=X_{i}-X_{i}$, *where* i∈〈1,2〉.
(b) *Cross-difference*: $D_{X-cd}=X_{i}-X_{j}$, *where* i∈〈1,2〉,j∈〈1,2〉, *while* i≠j.
(c) *Self-sum*: $S_{X-ss}=X_{i}+X_{i}$, *where* i∈〈1,2〉.
(d) *Cross-sum*: $S_{X-cs}=X_{i}+X_{j}$*, where* i∈〈1,2〉,j∈〈1,2〉*, while* i≠j.

**Definition 2.**  *(Co-array). For a linear sparse array* $\mathbb{P}=\left\{p_{i}|i\in \langle 1,G\rangle \right\}$*, one can define the following concepts:*

(a) *Difference co-array*: $D_{\mathbb{P}}=\mathbb{P}-\mathbb{P}=\left\{p_{i}-p_{j}|i\in \langle 1,G\rangle ,j\in \langle 1,G\rangle \right\}$.
(b) *Sum co-array*: $S_{\mathbb{P}}=\pm \left(\mathbb{P}+\mathbb{P}\right)=\left\{\pm \left(p_{i}+p_{j}\right)|i\in \langle 1,G\rangle ,j\in \langle 1,G\rangle \right\}$.
(c) *Difference–sum co-array*: $L_{\mathbb{P}}=D_{\mathbb{P}}\cup S_{\mathbb{P}}$.

Using $L_{\mathbb{P}}$, we can calculate the number of degrees of freedom as $\mathrm{DOFs}=\left|L_{\mathbb{P}}\right|$, the DOF ratio as $\gamma \left(G\right)=G^{2}/\left|L_{\mathbb{P}}\right|$ and the virtual aperture as $L=\max\left(L_{\mathbb{P}}\right)$, respectively. Note that $\left|L_{\mathbb{P}}\right|\geq \left|D_{\mathbb{P}}\right|$ when G≥2. As a result, the DSCA of a sparse array has more DOFs than the DCA, which has the potential to identify more sources.

### 3.1. Review of GNA-DCA

**Figure 1:**
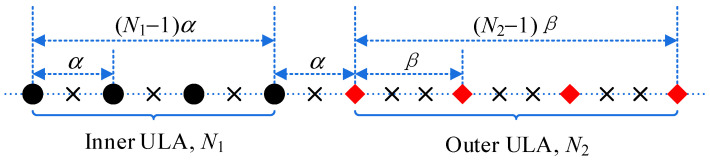
Generalized nested array, where we assume $N_{1}=N_{2}=4$. Black circles and red diamonds denote the inner ULA and outer ULA, respectively. Black crosses imply empty space when α>1,β>1.

As shown in Figure 1, a GNA, with $G=N_{1}+N_{2}$ sensors, is composed of two cascaded subarrays. The inner ULA has $N_{1}$ sensors with spacing α and the outer is a ULA of $N_{2}$ sensors with spacing β, where the spacing of the adjacent ULA is α. Then, the sensor locations, consisting of two integer sets, are collected as

(15) $$\{\begin{array}{l} \mathbb{P}={\mathbb{P}}_{1}\cup {\mathbb{P}}_{2}\\ {\mathbb{P}}_{1}=\left\{\alpha n_{1}|n_{1}\in \langle 0,N_{1}-1\rangle \right\}\\ {\mathbb{P}}_{2}=\left\{\alpha N_{1}+\beta n_{2}|n_{2}\in \langle 0,N_{2}-1\rangle \right\} \end{array}$$

where ${\mathbb{P}}_{1}$ and ${\mathbb{P}}_{2}$ denote the sensor locations of the inner ULA and outer ULA, respectively. α and β are a pair of integers.

**Lemma 1.**  *In the GNA, the DCA is generated only by vectorizing the covariance matrix of the array output. Hence, the following properties hold for the GNA-DCA* [20]:

(a) *When*$\alpha \in \langle 1,N_{2}\rangle ,\beta \in \langle 1,N_{1}+1\rangle$*, the range of positive and negative consecutive lags can be presented as*$\langle l_{1},l_{2}\rangle$*and*$\langle -l_{2},-l_{1}\rangle$*, respectively, where*$l_{1}=\left(\alpha -1\right)\left(\beta -1\right)$*and*$l_{2}=\alpha N_{1}+\beta N_{2}-\alpha \beta +\alpha -1$.
(b) *If*$\alpha \in \langle 1,N_{2}\rangle ,\beta \in \langle 1,N_{1}+1\rangle$*, the number of unique lags reaches*2l+1*, where*$l=\alpha N_{1}+\beta N_{2}-\alpha \beta +\alpha -1$.
(c) *In the case*$\alpha \in \langle 1,N_{2}\rangle ,\beta =N_{1}+1$*or*$\alpha =N_{2},\beta \in \langle 1,N_{1}+1\rangle$*, the maximum DOFs can be obtained, as detailed inTable 1*.

### 3.2. Proposed GNA-DSCA

In this subsection, we propose the generalized nested array via the difference–sum co-array (GNA-DSCA), which, unlike the GNA-DCA, which only considers the DCA, is a union of the DCA and the SCA. The closed-form expressions for the dilation factors and the number of DOFs are derived by analyzing both the DCA and SCA. Finally, the optimal physical configuration and the maximum DOFs are determined by solving a linear optimization problem. Since the GNA-DSCA can provide more DOFs and virtual aperture than the GNA-DCA, it can be used to achieve DOA estimation with superior performance.

Based on Definition 2 and Equation (15), the GNA-DSCA can be readily given by the following definition.

**Definition 3.**  *(GNA-DSCA). For a generalized nested array* $\mathbb{P}=\left\{p_{i}|i\in \langle 1,G\rangle \right\}$*, the DSCA can be defined as*

(16) $$\{\begin{array}{l} L_{\mathbb{P}}=L_{\mathbb{P}}^{+}\cup L_{\mathbb{P}}^{-}\\ L_{\mathbb{P}}^{+}=D_{\mathbb{P}}^{+}\cup S_{\mathbb{P}}^{+},L_{\mathbb{P}}^{-}=D_{\mathbb{P}}^{-}\cup S_{\mathbb{P}}^{-} \end{array}$$

*where*$L_{\mathbb{P}}^{+}$*and*$L_{\mathbb{P}}^{-}$*denote the positive and negative halves of*$L_{\mathbb{P}}$*, respectively.*$D_{\mathbb{P}}^{+}=\left\{p_{i}-p_{j}|i\in \langle 1,G\rangle ,j\in \langle 1,G\rangle ,i\geq j\right\}$*,*$S_{\mathbb{P}}^{+}=\left\{p_{i}+p_{j}|i\in \langle 1,G\rangle ,j\in \langle 1,G\rangle \right\}$. $D_{\mathbb{P}}^{-}$*and*$S_{\mathbb{P}}^{-}$*are their mirrored versions, i.e.,*$D_{\mathbb{P}}=D_{\mathbb{P}}^{+}\cup D_{\mathbb{P}}^{-}$*and*$S_{\mathbb{P}}=S_{\mathbb{P}}^{+}\cup S_{\mathbb{P}}^{-}$*. Clearly,*$L_{\mathbb{P}}$*is symmetric, i.e.,*$L_{\mathbb{P}}^{+}=-L_{\mathbb{P}}^{-}$*, where*$0\in L_{\mathbb{P}}^{+}$*and*$0\in L_{\mathbb{P}}^{-}$.

**Remark 1.**  *From Definition 2 and Definition 3, we can well explain that* $D_{\mathbb{P}}$ *and* $S_{\mathbb{P}}$ *are two incomplete equal sets and both of them are the subsets of* $L_{\mathbb{P}}$ *. Therefore, compared with the GNA-DCA, the GNA-DSCA can also obtain DOFs from the SCA, which provides the capacities to detect more sources.*

Next, we present some properties of the GNA-DSCA and provide rigorous proofs.

**Lemma 2.**  *Based on Definition 3, the properties of* $L_{\mathbb{P}}$ *can be derived as below:*

(a) *Provided that*$\alpha \in \langle 1,N_{2}\rangle ,\beta \in \langle 2,2N_{1}+1\rangle$*,*$L_{\mathbb{P}}^{+}$*possesses all the consecutive lags in the range*$\langle g_{1},g_{2}\rangle$*, where*$g_{1}=\left(\alpha -1\right)\left(\beta -1\right)$*and*$g_{2}=2\alpha N_{1}+\beta N_{2}-\alpha \beta +\alpha -1$*. Provided that*$\alpha \in \langle 1,N_{2}\rangle ,\beta =1$*,*$L_{\mathbb{P}}^{+}$*enjoys all the consecutive lags in the range*$\langle 0,2\left(\alpha N_{1}+N_{2}-1\right)\rangle$.
(b) *In the case*$\alpha \in \langle 1,N_{2}\rangle ,\beta \in \langle 1,2N_{1}+1\rangle$*, the total number of unique lags in*$L_{\mathbb{P}}$*reaches*2g−1*, where*$g=2\alpha N_{1}+\left(\beta +1\right)N_{2}-\alpha \beta +\alpha -1$.

**Proof.**  See Appendix A. □

**Remark 2.**  *Based on the proof of Lemma 2, we know that the number of inconsecutive lags in*$L_{\mathbb{P}}^{+}$*is*$\kappa_{1}+\kappa_{3}+\kappa_{4}=\left(\alpha -1\right)\left(\beta -1\right)+N_{2}-1$*, where*$\alpha \in \langle 1,N_{2}\rangle ,\beta \in \langle 2,2N_{1}+1\rangle$*. Thus, when*α=1*, the number of inconsecutive lags equals*$N_{2}-1$*, and all of them are distributed in the segment*$\Gamma_{4}$*. Inconsecutive lags also exist in the segment*$\Gamma_{1}$*and*$\Gamma_{3}$*when*α≠1*, which increase as the dilation factors*α*and*β*increase. Moreover, in the case*$\alpha \in \langle 1,N_{2}\rangle ,\beta =1$*, all the virtual sensors in*$L_{\mathbb{P}}^{+}$*are consecutive lags, which increase with the increase of*α.

In order to determine the maximum DOFs of the GNA-DSCA, we solve the closed-form expressions for g by choosing the optimal α and β. The optimization model is developed as

(17) $$\begin{array}{ll} \underset{\alpha ,\beta }{\max}\; & g=2\alpha N_{1}+\left(\beta +1\right)N_{2}-\alpha \beta +\alpha -1\\ \mathrm{s.t.} & 1\leq \alpha \leq N_{2},\\ & 1\leq \beta \leq 2N_{1}+1,\\ & G=N_{1}+N_{2} \end{array}$$

The solution of Equation (17) is $g_{\max}=2N_{1}N_{2}+2N_{2}-1$ , where $\;\alpha \in \langle 1,N_{2}\rangle ,\beta =2N_{1}+1$ or $\alpha =N_{2},\beta \in \langle 1,2N_{1}+1\rangle$.

**Proof.**  See Appendix B. □

Consequently, the optimal configuration and maximum DOFs obtained using the Arithmetic Mean–Geometric Mean (AM-GM) inequalities are summarized in Table 2.

**Remark 3.**  *Compared with the GNA-DCA, the GNA-DSCA has the following superior properties: (1) The dilation factor* β *in the GNA-DSCA can take more values, which means that the GNA-DSCA has more flexible physical configurations. (2) The larger* β *can further weaken the mutual coupling, thus making the GNA-DSCA more favorable for practical applications. (3) The GNA-DSCA keeps the virtue of a simple closed-form expression of DOFs. (4) The GNA-DSCA provides about twice as many maximum DOFs as the GNA-DCA with the same number of sensors. (5) The GNA-DSCA can further extend the virtual aperture using the sum co-array.*

**Figure 2:**
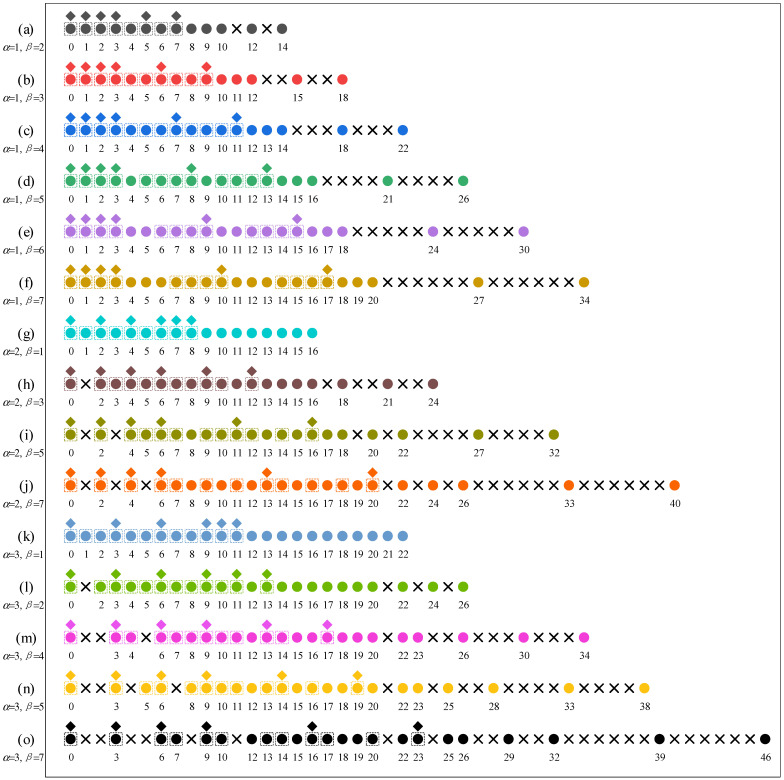
A family of six-sensor generalized nested arrays and their unique lags of the GNA-DCA and GNA-DSCA. Diamonds denote the sensor locations. Squares and circles indicate the unique lags in the GNA-DCA and GNA-DSCA, respectively, while crosses denote the hole locations.

To visualize the unique lags in the GNA-DCA and the GNA-DSCA, a family of six-sensor generalized nested arrays is considered in Figure 2, where α and β can take various values. We can see that the GNA-DSCA has more DOFs and a larger virtual aperture than the NA-DCA for any physical configuration. In addition, provided that β>4, the GNA-DCA no longer satisfies Lemma 1, while the GNA-DSCA meets Lemma 2. Therefore, the GNA-DSCA has more flexible array configurations.

**Table 2:** Optimal configuration and maximum DOFs for GNA-DSCA.

$G=N_{1}+N_{2}$	$\mathrm{Optimal}\;N_{1},N_{2}$	Optimal α,β	Maximum DOFs
Even	$N_{1}=N_{2}=G/2$	$\alpha \in \langle 1,N_{2}\rangle ,\beta =2N_{1}+1$ or $\alpha =N_{2},\beta \in \langle 1,2N_{1}+1\rangle$	$G^{2}+2G-3$
Odd	$N_{1}=\left(G-1\right)/2,N_{2}=\left(G+1\right)/2$		$G^{2}+2G-2$

## 4. DOA Estimation

In this section, we elaborate an efficient DOA estimation algorithm for the GNA-DSCA. The core of the algorithm lies in the construction of a Toeplitz matrix for two different cases of the observed vectors, which can be explained by the dilation factors. In particular, when α and β take larger values, we need to first fill additional virtual sensors at the hole locations and then recover the equivalent received signals based on the atomic norm minimization theory, so that we can obtain a covariance matrix similar to the ULA and then perform DOA estimation using the MUSIC algorithm.

The virtual array, obtained by the DSCA of the physical sensors, has many repeated lags, corresponding to the non-unique steer vectors in C. Therefore, the redundant values in r must be averaged [43] and then sorted in ascending order by virtual array locations to obtain a new observed vector as

(18) $$\tilde{r}=\tilde{C}u=\sum_{k=1}^{K}\tilde{c}\left(\theta_{k}\right){\left|u_{k}\right|}^{4}$$

where $\tilde{r}\in {\mathbb{C}}^{\left(2g-1\right)\times 1}$.

Depending on the type of observed vector corresponding to the DSCA, we can divide it into two cases to construct the Toeplitz matrix for DOA estimation.

**Case 1.**  *Based on Remark 2, if*$\alpha \in \langle 1,N_{2}\rangle ,\beta =1$*, we can obtain a hole-free GNA-DSCA. In this case, the following Toeplitz matrix can be constructed by referring to* [29]

(19) $$\tilde{R}=\left[\begin{array}{llll} {\langle \tilde{r}\rangle }_{0} & {\langle \tilde{r}\rangle }_{-1} & \cdots & {\langle \tilde{r}\rangle }_{-\left(g-1\right)}\\ {\langle \tilde{r}\rangle }_{1} & {\langle \tilde{r}\rangle }_{0} & \cdots & {\langle \tilde{r}\rangle }_{-\left(g-1\right)+1}\\ \vdots & \vdots & \ddots & \vdots \\ {\langle \tilde{r}\rangle }_{g-1} & {\langle \tilde{r}\rangle }_{g-2} & \cdots & {\langle \tilde{r}\rangle }_{0} \end{array}\right]$$

*where*${\langle \tilde{r}\rangle }_{i},i\in \langle -\left(g-1\right),g-1\rangle$*denotes the*i+g*-th element in*$\tilde{r}$.

**Case 2.**  *When Case 1 does not hold, some holes appear in the DSCA, which more or less affect the estimation performance. Therefore, we need to transform the non-uniform virtual array into a virtual ULA by filling zero statistics at the hole locations, i.e., use a hole-filling strategy. Then, the observation vector* $\tilde{r}$ *is updated as*

(20) $${\tilde{r}}_{0}={\tilde{C}}_{0}u=\sum_{k=1}^{K}{\tilde{c}}_{0}\left(\theta_{k}\right){\left|u_{k}\right|}^{4}$$

*where* ${\tilde{r}}_{0}\in {\mathbb{C}}^{\left(2g-1+2h\right)\times 1}$ *with* 2h *represents the number of zero* *statistics filled.*

Then, the Toeplitz matrix is constructed using ${\tilde{r}}_{0}$ as follows

(21) $${\tilde{R}}_{0}=\left[\begin{array}{cccc} {\langle {\tilde{r}}_{0}\rangle }_{0} & {\langle {\tilde{r}}_{0}\rangle }_{-1} & \cdots & {\langle {\tilde{r}}_{0}\rangle }_{-\left(g-1\right)-h}\\ {\langle {\tilde{r}}_{0}\rangle }_{1} & {\langle {\tilde{r}}_{0}\rangle }_{0} & \cdots & {\langle {\tilde{r}}_{0}\rangle }_{-\left(g-1\right)-h+1}\\ \vdots & \vdots & \vdots & \vdots \\ {\langle {\tilde{r}}_{0}\rangle }_{\left(g-1\right)+h} & {\langle {\tilde{r}}_{0}\rangle }_{\left(g-1\right)+h-1} & \cdots & {\langle {\tilde{r}}_{0}\rangle }_{0} \end{array}\right]$$

where ${\langle {\tilde{r}}_{0}\rangle }_{i},i\in \langle -\left(g-1\right)-h,\left(g-1\right)+h\rangle$ denotes the i+g+h-th element in ${\tilde{r}}_{0}$. Since ${\tilde{r}}_{0}$ contains several filled zero statistics, the diagonal entries at the corresponding positions in ${\tilde{R}}_{0}$ are also zero statistics.

Meanwhile, a binary vector $z\in {\mathbb{R}}^{\left(2g-1+2h\right)\times 1}$ is defined to distinguish the original lags from the filled zero statistics, i.e., the original lags correspond to 1 and the filled zero statistics correspond to 0, respectively. Next, we discuss the equivalent recovery of the zero statistics. The atomic set is first defined as follows

(22) $$A=\left\{{\tilde{c}}_{0}^{1}\left(\theta_{k}\right)|\theta_{k}\in \left[-\pi /2,\pi /2\right)\right\}$$

where ${\tilde{c}}_{0}^{1}\left(\theta_{k}\right)\in {\mathbb{C}}^{\left(g+h\right)\times 1}$ is formed by picking the g+h-th row to the 2g−1+2h-th row in ${\tilde{c}}_{0}\left(\theta_{k}\right)$.

The atomic norm of ${\tilde{r}}_{0}^{1}$ is defined as

(23) $${\left\|{\tilde{r}}_{0}^{1}\right\|}_{A}=\inf\left\{\sum_{k=1}^{K}p_{k}|{\tilde{r}}_{0}^{1}=\sum_{k=1}^{K}{\tilde{c}}_{0}^{1}(\theta_{k})p_{k},p_{k}>0\right\}$$

where inf{·} represents the infimum. ${\tilde{r}}_{0}^{1}\in {\mathbb{C}}^{\left(g+h\right)\times 1}$ is constructed by selecting the g+h-th row to the 2g−1+2h-th row in ${\tilde{r}}_{0}$.

Since the filled zero statistics actually do not receive sources, we can find a positive semi-definite (PSD) Hermitian Toeplitz matrix $T\left({\tilde{r}}_{0}^{1}\right)$ with the smallest difference from the corresponding elements of ${\tilde{R}}_{0}$ as the reconstructed covariance matrix; thus, the optimization model based on ANM is established as

(24) $$\begin{array}{ll} \underset{{\tilde{r}}_{0}^{1}\in {\mathbb{C}}^{g+h}}{\min} & \;{\left\|{\tilde{r}}_{0}^{1}\right\|}_{A}\\ \;\mathrm{s.t.} & {\left\|T\left({\tilde{r}}_{0}^{1}\right)⊕Z-{\tilde{R}}_{0}\right\|}_{F}^{2}\leq \delta ,\\ & T\left({\tilde{r}}_{0}^{1}\right)\geq 0 \end{array}$$

where $Z=zz^{T}$ is a binary matrix, which is used to distinguish the non-zero (original) and zero (filled) elements in ${\tilde{R}}_{0}$. δ is a threshold.

Using Lagrange multipliers, Equation (24) can be further transformed as

(25) $$\begin{array}{ll} \underset{{\tilde{r}}_{0}^{1}\in {\mathbb{C}}^{g+h}}{\min} & \;\;\frac{1}{2}\;{\left\|T\left({\tilde{r}}_{0}^{1}\right)⊕Z-{\tilde{R}}_{0}\right\|}_{F}^{2}+\zeta {\left\|{\tilde{r}}_{0}^{1}\right\|}_{A}\\ \;\mathrm{s.t.} & T\left({\tilde{r}}_{0}^{1}\right)\geq 0 \end{array}$$

where ζ is a regularization parameter.

Base on the PSD identity of $T\left({\tilde{r}}_{0}^{1}\right)$, in the case $\mathrm{rank}\left(T\left({\tilde{r}}_{0}^{1}\right)\right)<g+h$, $T\left({\tilde{r}}_{0}^{1}\right)$ can be decomposed by Vandermond as

(26) $$T\left({\tilde{r}}_{0}^{1}\right)=\sum_{k=1}^{K}p_{k}{\tilde{c}}_{0}^{1}\left(\theta_{k}\right){\left[{\tilde{c}}_{0}^{1}\left(\theta_{k}\right)\right]}^{H}$$

It follows naturally that the trace of $T\left({\tilde{r}}_{0}^{1}\right)$ can be obtained as

(27) $$\mathrm{trance}\left(T\left({\tilde{r}}_{0}^{1}\right)\right)=\left(\eta +h+1\right)\sum_{k=1}^{K}p_{k}$$

Observing ${\left\|{\tilde{r}}_{0}^{1}\right\|}_{A}$ in Equation (23) and $\mathrm{trace}\left(T\left({\tilde{r}}_{0}^{1}\right)\right)$ in Equation (27), we have

(28) $${\left\|{\tilde{r}}_{0}^{1}\right\|}_{A}=\mathrm{trace}\left(T\left({\tilde{r}}_{0}^{1}\right)\right)/\left(g+h\right)$$

As a result, the ANM problem in Equation (25) can be equivalently expressed as

(29) $$\begin{array}{ll} \underset{{\tilde{r}}_{0}^{1}\in {\mathbb{C}}^{g+h}}{\min} & \frac{1}{2}\;{\left\|T\left({\tilde{r}}_{0}^{1}\right)⊕Z-{\tilde{R}}_{0}\right\|}_{F}^{2}+\varepsilon \times \mathrm{trace}\left(T\left({\tilde{r}}_{0}^{1}\right)\right)\\ \;\mathrm{s.t.} & T\left({\tilde{r}}_{0}^{1}\right)\geq 0 \end{array}$$

where ε=ζ/(g+h). Here, the convex optimization problem in Equation (29) can be easily solved by the CVX toolbox [44].

Up to now, we have obtained the Toeplitz matrices for the two cases, which are $\tilde{R}$ and $T\left({\tilde{r}}_{0}^{1}\right)$, respectively. Since $\tilde{R}$ and $T\left({\tilde{r}}_{0}^{1}\right)$ correspond to the reference virtual array (i.e., a ULA), existing DOA estimation algorithms, including MUSIC [10], ESPRIT [11] and the sparsity-based technique [30], can be incorporated into the virtual domain for DOA estimation. For example, here, we present the MUSIC spectrum of $T\left({\tilde{r}}_{0}^{1}\right)$ as

(30) $$P_{\mathrm{MUSIC}}\left(\theta \right)=\frac{1}{{\left[{\tilde{c}}_{0}^{1}\left(\theta \right)\right]}^{H}N_{T\left({\tilde{r}}_{0}^{1}\right)}N_{T\left({\tilde{r}}_{0}^{1}\right)}^{H}{\tilde{c}}_{0}^{1}\left(\theta \right)}$$

where $N_{T\left({\tilde{r}}_{0}^{1}\right)}$ denotes the noise subspace of $T\left({\tilde{r}}_{0}^{1}\right)$. Note that the MUSIC spectrum of $\tilde{R}$ can be obtained in the same way.

It is important to note that Case 2 can recover the information contained in the missing lags, which is equivalent to improving the number of DOFs. Therefore, Case 2 has a better estimation performance than Case 1, which only utilizes the consecutive lags.

## 5. Simulation Results

In this section, four sets of numerical simulations involving virtual array properties, MUSIC spectra, Root-Mean-Square Error (RMSE) and resolution performance are conducted to evaluate the superiority of the GNA-DSCA. Several recently proposed configurations, including the NA-DCA [15], CPA-DCA [16], GNA-DCA [20], NA-DSCA [41], CPA-DSCA [40], DsNA [41], INAwSDCA−I [42] and INAwSDCA−II [42], are provided for comparison. The peak search range for the MUSIC spectra is from −90° to 90° with a small grid of 0.01°.

### 5.1. Virtual Array Properties

**Figure 3:**
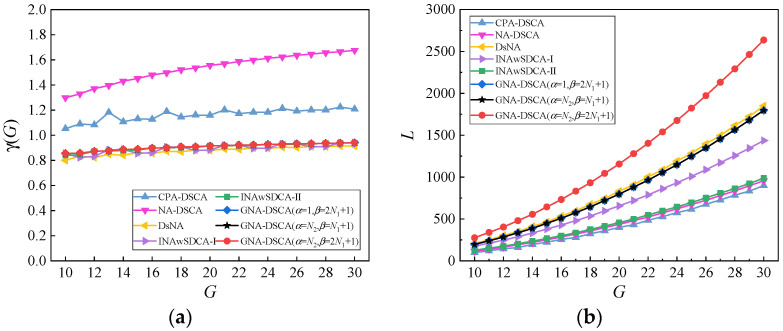
Comparison of virtual array properties. (**a**) DOF ratio γ(G) versus the number of sensors G; (**b**) Virtual aperture L versus the number of sensors G.

In the underlying numerical simulations, the DOF ratio and virtual aperture for different DSCA-based sparse arrays are compared in Figure 3a,b, respectively, where the number of sensors varies from 10 to 30. It is observed from Figure 3a that the NA-DSCA possesses the smallest DOFs, followed by the CPA-DSCA, while the GNA-DSCA, DsNA, INAwSDCA−I and INAwSDCA−II have the highest DOFs and basically remain at the same level. It is worth noting that the GNA-DSCA can significantly increase the DOFs than the NA-DSCA. This phenomenon is attributed to the dense subarray, which causes the NA-DSCA to have many redundant lags, while the GNA-DSCA uses the dilation factors to reduce the number of dense sensors, thus achieving more DOFs. It is also determined from Figure 3b that the order of the virtual aperture size corresponding to each configuration is: GNA-DSCA ($\alpha =N_{2},\beta =2N_{1}+1$) > DsNA > GNA-DSCA ($\alpha =1,\beta =2N_{1}+1$) = GNA-DSCA ($\alpha =N_{2},\beta =N_{1}+1$) > INAwSDCA−I > INAwSDCA−II > NA-DSCA > CPA-DSCA. By introducing the dilation factors, the GNA-DSCA can significantly enlarge the virtual aperture. Therefore, the inter-sensor spacing is an essential factor affecting the virtual aperture.

### 5.2. MUSIC Spectra

In the second numerical simulations, we evaluate the DOA estimation performance by presenting the MUSIC spectra of various sparse arrays specified in Table 3, where the coupling coefficients used in Refs. [17,25] are as follows: $c_{0}=1$, $c_{1}=0.3e^{J\pi /3}$ and $c_{l}=c_{1}e^{-J(l-1)\pi /8}/l,2\leq l\leq B,B=100$. Note that, when using the hole-filling strategy for DOA estimation, the virtual aperture is equivalent to the effective DOFs.

**Figure 4:**
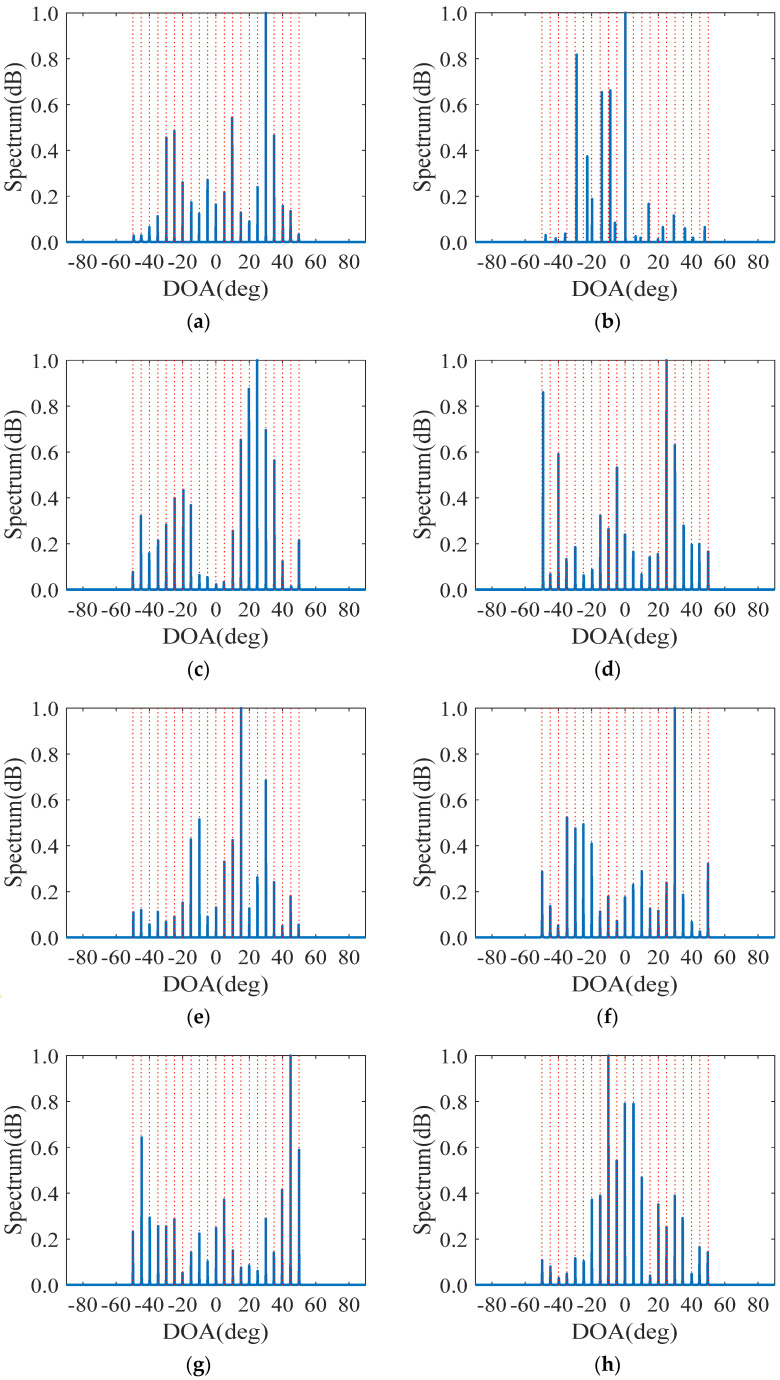

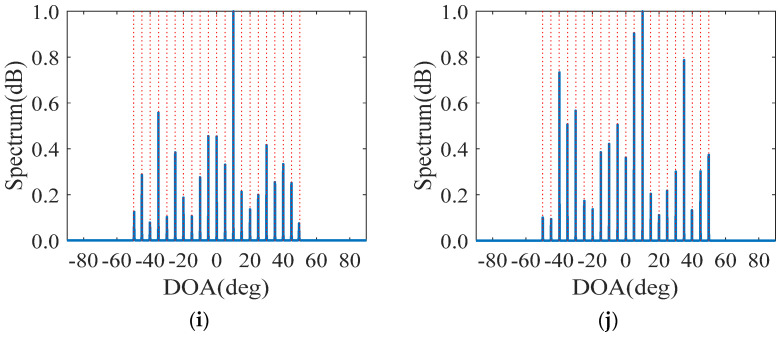
Comparison of normalized spatial spectra, where K=21, SNR=0 dB and $N_{p}=100$. The blue solid line and the red dashed line represent the estimation results and the actual angels, respectively. (**a**) NA-DCA; (**b**) CPA-DCA; (**c**) GNA-DCA (α=5,β=6); (**d**) CPA-DSCA; (**e**) DsNA; (**f**) INAwSDCA−I; (**g**) INAwSDCA−II; (**h**) GNA-DSCA (α=1,β=11); (**i**) GNA-DSCA (α=5,β=6); (**j**) GNA-DSCA (α=5,β=11).

Assume that there are K=21 far-field, narrowband and uncorrelated incident sources distributed from −50° to 50° with an interval of 5°. The normalized MUSIC spectra are depicted in Figure 4, where the input signal-to-noise ratio (SNR) is 0dB, and the number of pseudo-sampling snapshots is set to $N_{p}=100$. We can observe from Figure 4 that the CPA-DCA can only estimate 19 sources, and the individual results seriously deviate from the actual source angels. The reason lies in that the CPA-DCA only utilizes the smallest virtual aperture generated by the DCA, which has a detrimental effect on the spectra. In contrast, the GNA-DSCA and the other six configurations are capable of estimating all the 21 sources. Since the virtual aperture of the NA-DCA and GNA-DCA (α=5,β=6) are relatively small, their spectra are not as prominent as those of the DSCA-based sparse arrays. As a result, the sparse arrays based on the DSCA can effectively improve the DOA estimation performance.

### 5.3. Root-Mean-Square Error

To examine the accuracy of the GNA-DSCA in DOA estimation, we compare the RMSE of the 21 sources described in Section 5.2 through 200 Monte Carlo trials. The RMSE is calculated by

(31) $$\mathrm{RMSE}=\sqrt{\frac{1}{200\times 21}\sum_{i=1}^{200}\sum_{k=1}^{21}{\left({\hat{\theta }}_{k}^{i}-\theta_{k}\right)}^{2}}$$

where ${\hat{\theta }}_{k}^{i}$ represents the estimated DOA of $\theta_{k}$ in the i-th Monte Carlo trial.

**Figure 5:**
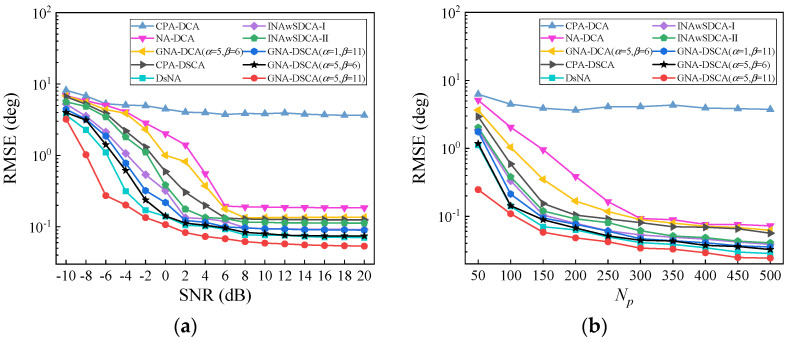
Comparison of RMSE, where K=21. (**a**) RMSE versus SNR with $N_{p}=100$; (**b**) RMSE versus the number of pseudo-sampling snapshots with SNR=0 dB.

The RMSE curves as a function of the SNR and the number of pseudo-sampling snapshots are depicted in Figure 5, where we assume $N_{p}=100$ for Figure 5a and SNR=0 dB for Figure 5b. We can observe from Figure 5a that the estimation accuracy of all configurations improves with the increasing SNR. The RMSE curves of all configurations begin to level off when SNR≥6 dB. For each RMSE point, the GNA-DSCA (α=5,β=11) outperforms the others, while the CPA-DCA has the worst performance. Noteworthily, although the three GNA-DSCA configurations have the same number of DOFs, the GNA-DSCA (α=5,β=11) has the best estimation performance over the GNA-DSCA (α=1,β=11) and GNA-DSCA (α=5,β=6). The reason for this phenomenon is that the GNA-DSCA (α=5,β=11) can generate a larger virtual aperture by using the larger dilation factors, which can be considered an equivalent increase in the number of DOFs when the hole-filling strategy is adopted for DOA estimation. Moreover, compared with the DCA-based sparse arrays, the DSCA-based sparse arrays can exploit the DOFs contained in the SCA; thus, they have a superior estimation performance. Therefore, the accuracy of DOA estimation is positively relevant to the number of DOFs. Similar estimation results can also be observed in Figure 5b, where the RMSE curves of all configurations tend to be flat when $N_{p}\geq 300$.

### 5.4. Resolution Performance

In the fourth numerical simulations, we compare the resolution performance by estimating two incident sources within a 3dB beam width, which is defined as

(32) $$2\theta_{0.5}=2\sin^{-1}\left(\frac{1.4\lambda }{\pi Ld}\right)=2\sin^{-1}\left(\frac{2.8}{L\pi }\right)$$

where L denotes the available virtual aperture for DOA estimation. From Table 3, the virtual aperture of the GNA-DSCA (α=5,β=11) equals 138, which leads to its 3 dB beam width of about 0.740°.

**Figure 6:**
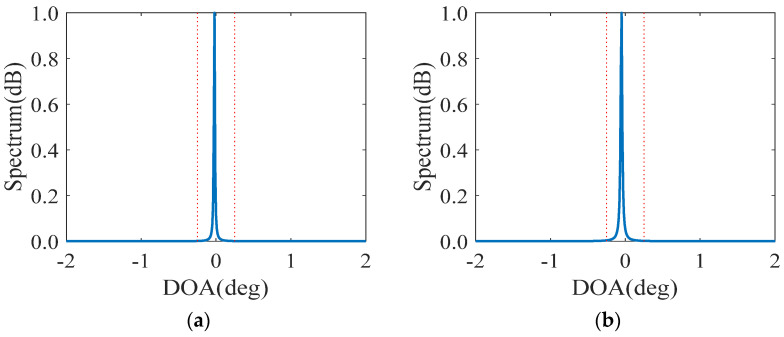

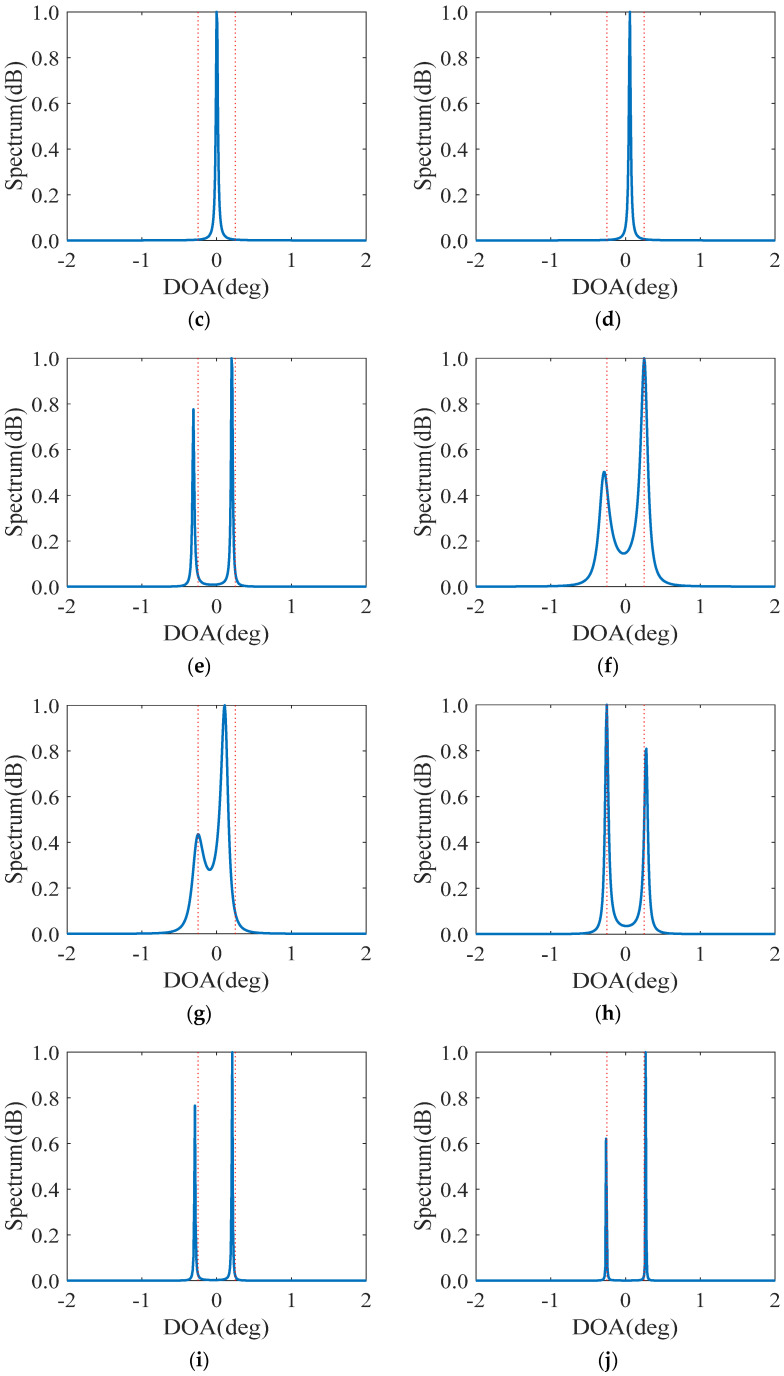
Comparison of resolution, where K=2, SNR=0 dB and $N_{p}=100$. The blue solid line and the red dashed line represent the estimation results and the actual angels, respectively. (**a**) NA-DCA; (**b**) CPA-DCA; (**c**) GNA-DCA (α=5,β=6); (**d**) CPA-DSCA; (**e**) DsNA; (**f**) INAwSDCA−I; (**g**) INAwSDCA−II; (**h**) GNA-DSCA (α=1,β=11); (**i**) GNA-DSCA (α=5,β=6); (**j**) GNA-DSCA (α=5,β=11).

Figure 6 depicts the normalized spatial spectra, where two close sources are located at −0.25° and 0.25° with SNR=0 dB and $N_{p}=100$. We can observe from Figure 6 that the DCA-based sparse arrays and the CPA-DSCA fail to identify the two incident sources within a 3 dB beam width. In contrast, other DSCA-based sparse arrays achieve accurate super-resolution estimation, which is mainly attributed to the larger available virtual aperture. Since a larger virtual aperture can be obtained in the DsNA and GNA-DSCA, their normalized spatial spectra, plotted in Figure 6e,h,i,j, have sharper peaks and more precise estimates than the INAwSDCA−I and INAwSDCA−II. As a result, the larger the virtual aperture is, the higher the resolution is.

## 6. Conclusions

In this paper, we consider the DSCA involving both the temporal and spatial information of the received signal to conduct DOA estimation. We first investigate the generalized nested array based on the DSCA (GNA-DSCA), where the closed-form expression for DOFs is derived as a function of the dilation factors and the number of sensors. The best results, including the dilation factors and DOFs, are presented to exhibit the excellent properties of the GNA-DSCA. Then, a hole-filling strategy is introduced to deal with the virtual array discontinuity caused by the larger dilation factors. Finally, DOA estimation is performed based on the reconstructed Toeplitz matrix. The superiorities of the GNA-DSCA are verified by extensive numerical simulations.

## Figures and Tables

**Table 1:** Optimal configuration and maximum DOFs for GNA-DCA.

$G=N_{1}+N_{2}$	$\mathrm{Optimal}\;N_{1},N_{2}$	Optimal α,β	Maximum DOFs
Even	$N_{1}=N_{2}=G/2$	$\alpha \in \langle 1,N_{2}\rangle ,\beta =N_{1}+1$ or $\alpha =N_{2},\beta \in \langle 1,N_{1}+1\rangle$	$\left(G^{2}-2\right)/2+G$
Odd	$N_{1}=\left(G-1\right)/2,N_{2}=\left(G+1\right)/2$		$\left(G^{2}-1\right)/2+G$

**Table 3:** Comparisons of configuration, DOFs, virtual aperture and coupling leakage; number of sensors G=10.

Sparse Array	Configuration	DOFs	Virtual Aperture	Coupling Leakage
NA-DCA	{0, 1, 2, 3, 4, 5, 11, 17, 23, 29}	59	29	0.5022
CPA-DCA	{0, 5, 6, 10, 12, 15, 18, 20, 24, 25}	39	25	0.2929
GNA-DCA (α=5,β=6)	{0, 5, 10, 15, 20, 25, 31, 37, 43, 49}	59	49	0.1215
CPA-DSCA	{0, 5, 6, 10, 12, 15, 18, 20, 24, 25}	95	50	0.2929
DsNA	{0, 3, 5, 7, 8, 17, 26, 35, 44, 53}	125	106	0.3187
INAwSDCA−I	{0, 16, 17, 18, 19, 20, 26, 32, 38, 44}	117	88	0.4806
INAwSDCA−II	{0, 9, 15, 21, 27, 28, 29, 30, 31, 32}	119	64	0.5022
GNA-DSCA (α=1,β=11)	{0, 1, 2, 3, 4, 5, 16, 27, 38, 49}	117	98	0.4991
GNA-DSCA (α=5,β=6)	{0, 5, 10, 15, 20, 25, 31, 37, 43, 49}	117	98	0.1215
GNA-DSCA (α=5,β=11)	{0, 5, 10, 15, 20, 25, 36, 47, 58, 69}	117	138	0.1126

**Figure A1:**
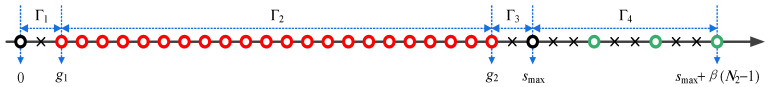
The distributions of the unique lags in the positive set $L_{\mathbb{P}}^{+}$, where circles and crosses denote the unique lags and holes, respectively.

## Data Availability

The data that support the findings of this research are available from the corresponding author.

## Appendix A. Proof of Lemma 2

(a) For Lemma 2(a), since $L_{\mathbb{P}}^{+}=D_{\mathbb{P}}^{+}\cup S_{\mathbb{P}}^{+}$, we need to analyze the elements in $D_{\mathbb{P}}^{+}$ and $S_{\mathbb{P}}^{+}$, respectively. The set $D_{\mathbb{P}}^{+}$ can be expressed as [20]

(A1)
$$D_{\mathbb{P}}^{+}=\left\{\alpha n_{1}+\beta n_{2}|n_{1}\in \langle 0,N_{1}\rangle ,n_{2}\in \langle 0,N_{2}-1\rangle \right\}$$

Since $S_{\mathbb{P}}^{+}$ is the union of self-sum $S_{\mathbb{P}-ss}^{+}$ and cross-sum $S_{\mathbb{P}-cs}^{+}$, it can be given by

(A2) $$\{\begin{array}{l} S_{\mathbb{P}}^{+}=S_{\mathbb{P}-ss}^{+}\cup S_{\mathbb{P}-cs}^{+}\\ S_{\mathbb{P}-ss}^{+}=\left({\mathbb{P}}_{1}+{\mathbb{P}}_{1}\right)\cup \left({\mathbb{P}}_{2}+{\mathbb{P}}_{2}\right)\\ S_{\mathbb{P}-cs}^{+}=\left({\mathbb{P}}_{1}+{\mathbb{P}}_{2}\right) \end{array}$$

where

(A3) $${\mathbb{P}}_{1}+{\mathbb{P}}_{1}=\left\{\alpha n_{1}|n_{1}\in \langle 0,2\left(N_{1}-1\right)\rangle \right\}$$

(A4) $${\mathbb{P}}_{2}+{\mathbb{P}}_{2}=\left\{2\alpha N_{1}+\beta n_{2}|n_{2}\in \langle 0,2\left(N_{2}-1\right)\rangle \right\}$$

(A5) $$\begin{array}{cc} {\mathbb{P}}_{1}+{\mathbb{P}}_{2} & =\left\{\alpha N_{1}+\alpha n_{1}+\beta n_{2}|n_{1}\in \langle 0,N_{1}-1\rangle ,n_{2}\in \langle 0,N_{2}-1\rangle \right\}\\ & =\left\{\alpha n_{1}+\beta n_{2}|n_{1}\in \langle N_{1},2N_{1}-1\rangle ,n_{2}\in \langle 0,N_{2}-1\rangle \right\} \end{array}$$

According to Equations (16) and (A1)–(A5), the set $L_{\mathbb{P}}^{+}$ can be specified as

(A6) $$\begin{array}{cc} L_{\mathbb{P}}^{+} & =D_{\mathbb{P}}^{+}\cup S_{\mathbb{P}}^{+}=D_{\mathbb{P}}^{+}\cup S_{\mathbb{P}-cs}^{+}\cup S_{\mathbb{P}-ss}^{+}\\ & =\left(D_{\mathbb{P}}^{+}\cup S_{\mathbb{P}-cs}^{+}\cup \left({\mathbb{P}}_{1}+{\mathbb{P}}_{1}\right)\right)\cup \left({\mathbb{P}}_{2}+{\mathbb{P}}_{2}\right)\\ & =\left\{\alpha n_{1}+\beta n_{2}|n_{1}\in \langle 0,2N_{1}-1\rangle ,n_{2}\in \langle 0,N_{2}-1\rangle \right\}\cup \left\{2\alpha N_{1}+\beta n_{2}|n_{2}\in \langle 0,2\left(N_{2}-1\right)\rangle \right\}\\ & =\left\{\alpha n_{1}+\beta n_{2}|n_{1}\in \langle 0,2N_{1}-1\rangle ,n_{2}\in \langle 0,N_{2}-1\rangle \right\}\cup \left\{\alpha n_{1}+\beta n_{2}|n_{1}=2N_{1},n_{2}\in \langle 0,2\left(N_{2}-1\right)\rangle \right\}\\ & =\left\{\alpha n_{1}+\beta n_{2}|n_{1}\in \langle 0,2N_{1}\rangle ,n_{2}\in \langle 0,N_{2}-1\rangle \right\}\cup \left\{\alpha n_{1}+\beta n_{2}|n_{1}=2N_{1},n_{2}\in \langle 0,2\left(N_{2}-1\right)\rangle \right\}\\ & =L_{1}^{+}\cup L_{2}^{+} \end{array}$$

where $L_{1}^{+}=\left\{\alpha n_{1}+\beta n_{2}|n_{1}\in \langle 0,2N_{1}\rangle ,n_{2}\in \langle 0,N_{2}-1\rangle \right\}$, $L_{2}^{+}=\left\{\alpha n_{1}+\beta n_{2}|n_{1}=2N_{1},n_{2}\in \langle N_{2},2\left(N_{2}-1\right)\rangle \right\}$.

From Equation (A6), we know that the consecutive lags can only be generated in $L_{1}^{+}$ for the case $\alpha \in \langle 1,N_{2}\rangle ,\beta \in \langle 2,2N_{1}+1\rangle$. Hence, we need proof that there exist $n_{1}\in \langle 0,2N_{1}\rangle$ and $n_{2}\in \langle 0,N_{2}-1\rangle$, so that $s=\alpha n_{1}+\beta n_{2}$ traverses all the integers in the range $\langle g_{1},g_{2}\rangle$, where $g_{1}=\left(\alpha -1\right)\left(\beta -1\right)$ and $g_{2}=2\alpha N_{1}+\beta N_{2}-\alpha \beta +\alpha -1$.

The independent variable $n_{2}\in \langle 0,N_{2}-1\rangle$ can be reformulated as

(A7) $$0\leq \beta n_{2}\leq \beta \left(N_{2}-1\right)$$

Introducing $\alpha n_{1}=s-\beta n_{2}$ and $\left(\alpha -1\right)\left(\beta -1\right)\leq s\leq 2\alpha N_{1}+\beta N_{2}-\alpha \beta +\alpha -1$ into Equation (A7), we can obtain

(A8) $$\beta \left(\alpha -N_{2}\right)-\alpha +1\leq \alpha n_{1}\leq \beta \left(N_{2}-\alpha \right)+2\alpha N_{1}+\alpha -1$$

Since $\alpha \in \langle 1,N_{2}\rangle$, we have $\alpha -N_{2}\leq 0$ and $N_{2}-\alpha \geq 0$. Thus, Equation (A8) can be simplified as

(A9) $$-\alpha +1\leq \alpha n_{1}\leq 2\alpha N_{1}+\alpha -1$$

Dividing Equation (A9) by α yields

(A10) $$-1+1/\alpha \leq n_{1}\leq 2N_{1}+1-1/\alpha$$

Since 1/α≤1, Equation (A10) can be further expressed as $0\leq n_{1}\leq 2N_{1}$, which is satisfied with the set $L_{1}^{+}$.

Notice that if $\alpha \in \langle 1,N_{2}\rangle ,\beta =1$, we can easily prove that the range of consecutive lags in $L_{1}^{+}$ and $L_{2}^{+}$ are $\langle 0,2\alpha N_{1}+N_{2}-1\rangle$ and $\langle 0,2\alpha N_{1}+N_{2}-1\rangle$, respectively. Therefore, $L_{\mathbb{P}}^{+}$ enjoys all the consecutive lags in the range $\langle 0,2\left(\alpha N_{1}+N_{2}-1\right)\rangle$.

(b) For Lemma 2(b), we take the positive set $L_{\mathbb{P}}^{+}$ as an example. We need proof that $L_{\mathbb{P}}^{+}$ possesses g unique lags for the case $\alpha \in \langle 1,N_{2}\rangle ,\beta \in \langle 1,2N_{1}+1\rangle$, where $g=2\alpha N_{1}+\left(\beta +1\right)N_{2}-\alpha \beta +\alpha -1$.

The maximum lags in the set $L_{1}^{+}$ are calculated as

(A11) $$s_{\max}=2\alpha N_{1}+\beta \left(N_{2}-1\right)$$

In the case $\alpha \in \langle 1,N_{2}\rangle ,\beta \in \langle 2,2N_{1}+1\rangle$, the distributions of the unique lags in the positive set $L_{\mathbb{P}}^{+}$ are depicted in Figure A1, which is composed of four segments ($\Gamma_{1},\Gamma_{2},\Gamma_{3},\Gamma_{4}$). Based on Lemma 2(a), we can conclude that only $\Gamma_{2}$ is a continuous segment, while holes exist in $\Gamma_{1}$, $\Gamma_{3}$ and $\Gamma_{4}$. Thereby, the number of unique lags in the segment $\Gamma_{3}$ satisfies

(A12) $$\begin{array}{l} 0\leq \kappa_{3}\leq s_{\max}-g_{2}\\ \Rightarrow 0\leq \kappa_{3}\leq \left(\alpha -1\right)\left(\beta -1\right) \end{array}$$

Due to the coprime characteristic of α and β in [24], we can draw the conclusion that $\kappa_{3}=\left(\alpha -1\right)\left(\beta -1\right)/2$. Likewise, the number of unique lags in the segment $\Gamma_{1}$ equals $\kappa_{1}=\left(\alpha -1\right)\left(\beta -1\right)/2$. From Equation (A6), we can know the number of unique lags in the segment $\Gamma_{4}$ when $\kappa_{4}=N_{2}-1$.

Therefore, the number of unique lags in the positive set $L_{\mathbb{P}}^{+}$ is equal to

(A13) $$\begin{array}{ll} g & =g_{2}-g_{1}+1+\kappa_{1}+\kappa_{3}+\kappa_{4}\\ & =2\alpha N_{1}+\left(\beta +1\right)N_{2}-\alpha \beta +\alpha -1 \end{array}$$

Note that, if $\alpha \in \langle 1,N_{2}\rangle ,\beta =1$, the set $L_{\mathbb{P}}^{+}$ includes all the consecutive lags in the range $\langle 0,2\left(\alpha N_{1}+N_{2}-1\right)\rangle$. Thus, the number of unique lags in $L_{\mathbb{P}}^{+}$ equals

(A14) $$g=2\left(\alpha N_{1}+N_{2}-1\right)+1$$

In summary, in the case $\alpha \in \langle 1,N_{2}\rangle ,\beta \in \langle 1,2N_{1}+1\rangle$, the total number of unique lags in the set $L_{\mathbb{P}}^{+}$ reaches

(A15) $$g=2\alpha N_{1}+\left(\beta +1\right)N_{2}-\alpha \beta +\alpha -1$$

This completes the proof. □

## Appendix B. Solution of Equation (17)

The partial derivatives of g with respect to α and β can be calculated as

(A16) $$\{\begin{array}{l} \frac{\partial g}{\partial \alpha }=2N_{1}+1-\beta >0\\ \frac{\partial g}{\partial \beta }=N_{2}-\alpha \geq 0 \end{array}$$

We can observe from Equation (A16) that the function g increases monotonically, which indicates that the DOFs are the maximum when α or β take the maximum value. To further determine the exact value, g can be reformulated as

(A17) $$\begin{array}{cc} g & =2\alpha N_{1}+\left(\beta +1\right)N_{2}-\alpha \beta +\alpha -1\\ & =\alpha \left(2N_{1}+1-\beta \right)+\left(\beta +1\right)N_{2}-1\\ & =\beta \left(N_{2}-\alpha \right)+\alpha \left(2N_{1}+1\right)+N_{2}-1 \end{array}$$

We can see from Equations (A16) and (A17) that, when $\alpha =N_{2}$ or $\beta =2N_{1}+1$, g can always obtain the maximum value, i.e., $g_{\max}=2N_{1}N_{2}+2N_{2}-1$.

This completes the proof. □
